# Supplementary material for: Protease-activated receptor 2 deficient mice develop less angiotensin II induced left ventricular hypertrophy but more cardiac fibrosis
Source: PLoS One. 2024 Dec 5;19(12):e0310095. doi: 10.1371/journal.pone.0310095 (PMC11620577; doi:10.1371/journal.pone.0310095)
Supplement: S1 Table — (DOCX) [file pone.0310095.s001.docx]

**S1: Primer used for Real-time PCR**

| **gene** | **species** | **Forward primer 5‘-3‘** | **Reverse primer 5‘-3‘** |
| --- | --- | --- | --- |
| *18s* | mouse/rat | TTGATTAAGTCCCTGCCCTTTGT | CGATCCGAGGGCCTCACTA |
| *F2rl1* (PAR2) | mouse | CCGGACCGAGAACCTTG | CGGAAGAAAGACAGTGGTCAG |
| *Fgf23* | mouse | GTATGGATCTCCACGGCAAC | AGACGTCATAGCCATTCTCCA |
| *Col1a1* | mouse | TCACCTACAGCACCCTTGTGG | CCCAAGTTCCGGTGTGACTC |
| *Nppb* (BNP) | mouse | GAGGTCACTCCTATCCTCTGG | GCCATTTCCTCCGACTTTTCTC |
| *Tnnt2* (Cardiac troponin) | mouse | GCCCTCAAACTTTTTCTTTCGGA | CTGATGCTGCAGATTGCGAAG |
| *Tgfb1* (TGF-ß) | rat | TGGAAGTGGATCCACGCGCCCAAGG | GCAGGAGCGCACGATCATGTTGGAC |
| *Col1a1* | rat | TCACCTACAGCACGCTTG | GGTCTGTTTCCAGGGTTG |
| *Col4a1* | rat | AACGAAAGGGACACGAGGA | GGCCAGGAATACCAGGAAGT |
| *Acta2* (SMA) | rat | CGGGCTTTGCTGGTGATG | CCCACGATGGATGGGAAA |
| *Fn1* (Fibronectin) | rat | TTGCAACCCACCGTGGAGTATGTG | CTCGGTAGCCAGTGAGCTTAACAC |
