# Supplementary material for: Protease-activated receptor 2 deficient mice develop less angiotensin II induced left ventricular hypertrophy but more cardiac fibrosis
Source: PLoS One. 2024 Dec 5;19(12):e0310095. doi: 10.1371/journal.pone.0310095 (PMC11620577; doi:10.1371/journal.pone.0310095)
Supplement: S1 File — (DOCX) [file pone.0310095.s002.docx]

**S2: Detailed parameters used for the evaluation** **of P-ERK1/2 and P-Smad3-positive cardiac rat fibroblasts**

**Nucleus Parameters: CH1 (Dapi)**

Background Radius: 8um

Sigma: 4

Minimum area: 10um^2

Maximum area: 400um^2

**pERK:**

**Intensity Parameters:**

Threshold 10

Cell expansion: 5um

**Intensity threshold parameters:**

Score compartment: Nucleus: CH2-T2 max

Threshold 1+ (low positive intensity threshold): 60

Threshold 2+ (moderate positive intensity theshold): 20

Threshold 3+ (high positive intensity threshold): 30

runPlugin('qupath.imagej.detect.cells.PositiveCellDetection', '{"detectionImage":"Ch1-T1","requestedPixelSizeMicrons":0.5,"**backgroundRadiusMicrons":8.0,"**backgroundByReconstruction":true,"medianRadiusMicrons":0.0**,"sigmaMicrons":4.0**,"**minAreaMicrons":10.0**,"**maxAreaMicrons":400.0**,"**threshold":10.0**,"watershedPostProcess":true,"**cellExpansionMicrons":5.0**,"includeNuclei":true,"smoothBoundaries":true,"makeMeasurements":true,"thresholdCompartment":"**Nucleus**: **Ch2-T2max**","**thresholdPositive1":60.0**,"**thresholdPositive2":20.0**,"**thresholdPositive3":30.0**,"singleThreshold":true}')

**pSMAD:**

**Intensity Parameters:**

Threshold 25

Cell expansion: 5um

**Intensity threshold parameters:**

Score compartment: Nucleus: CH2-T2 max

Threshold 1+ (low positive intensity threshold): 90

Threshold 2+ (moderate positive intensity theshold): 20

Threshold 3+ (high positive intensity threshold): 30

runPlugin('qupath.imagej.detect.cells.PositiveCellDetection', '{"detectionImage":"Ch1-T1","requestedPixelSizeMicrons":0.5,"**backgroundRadiusMicrons":8.0**,"backgroundByReconstruction":true,"medianRadiusMicrons":0.0,"**sigmaMicrons":4.0**,"**minAreaMicrons":10.0**,"**maxAreaMicrons":400.0**,"**threshold":25.0**,"watershedPostProcess":true,"**cellExpansionMicrons":5.0**,"includeNuclei":true,"smoothBoundaries":true,"makeMeasurements":true,"thresholdCompartment":"**Nucleus:** **Ch2-T2 max**","**thresholdPositive1":90.0**,"**thresholdPositive2":20.0**,"**thresholdPositive3":30.0**,"singleThreshold":true}')
