# Supplementary figures and images for: Protease-activated receptor 2 deficient mice develop less angiotensin II induced left ventricular hypertrophy but more cardiac fibrosis
Source: PLoS One. 2024 Dec 5;19(12):e0310095. doi: 10.1371/journal.pone.0310095 (PMC11620577; doi:10.1371/journal.pone.0310095)

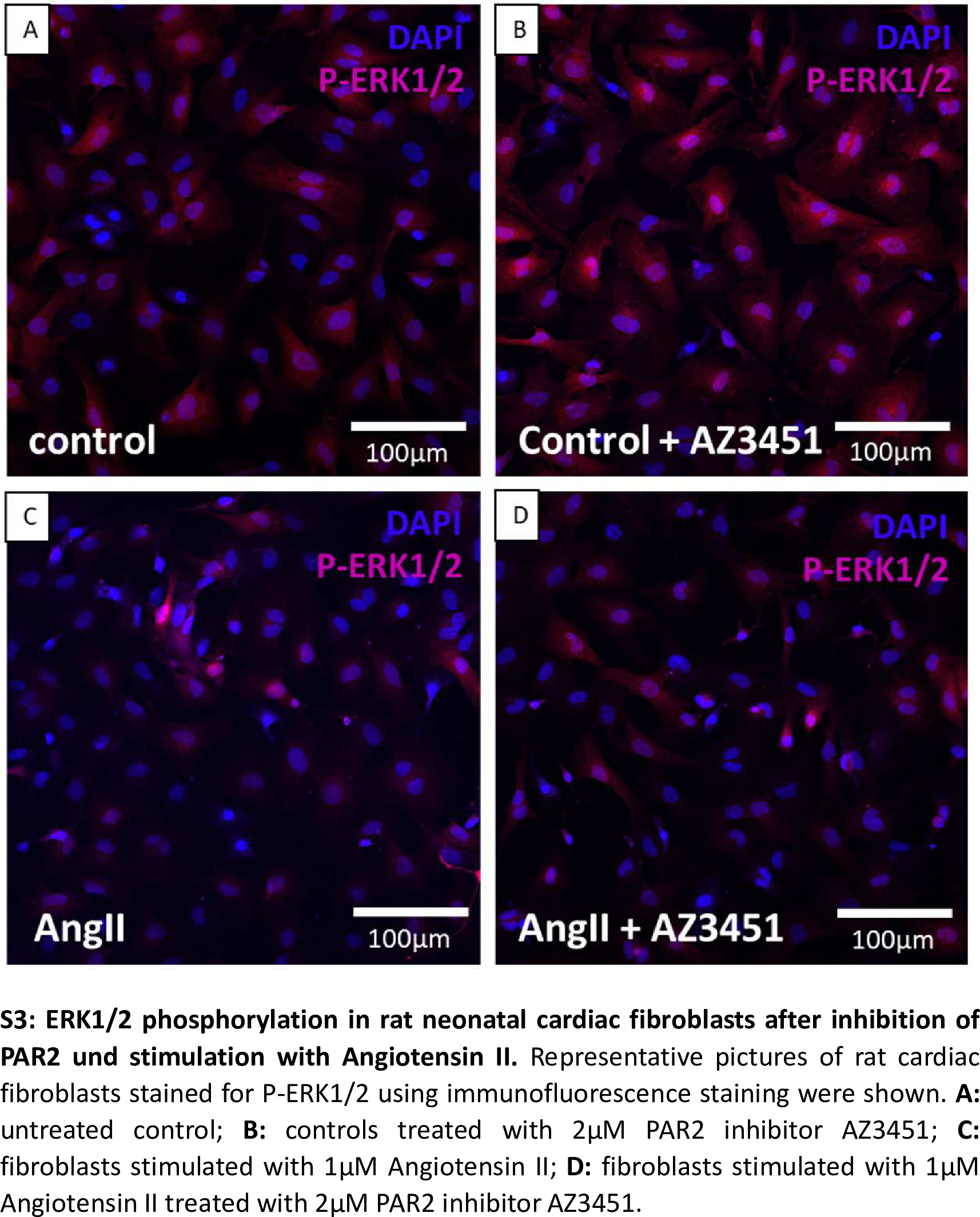

Supplement: S1 Fig — (TIF) [file pone.0310095.s003.tif]

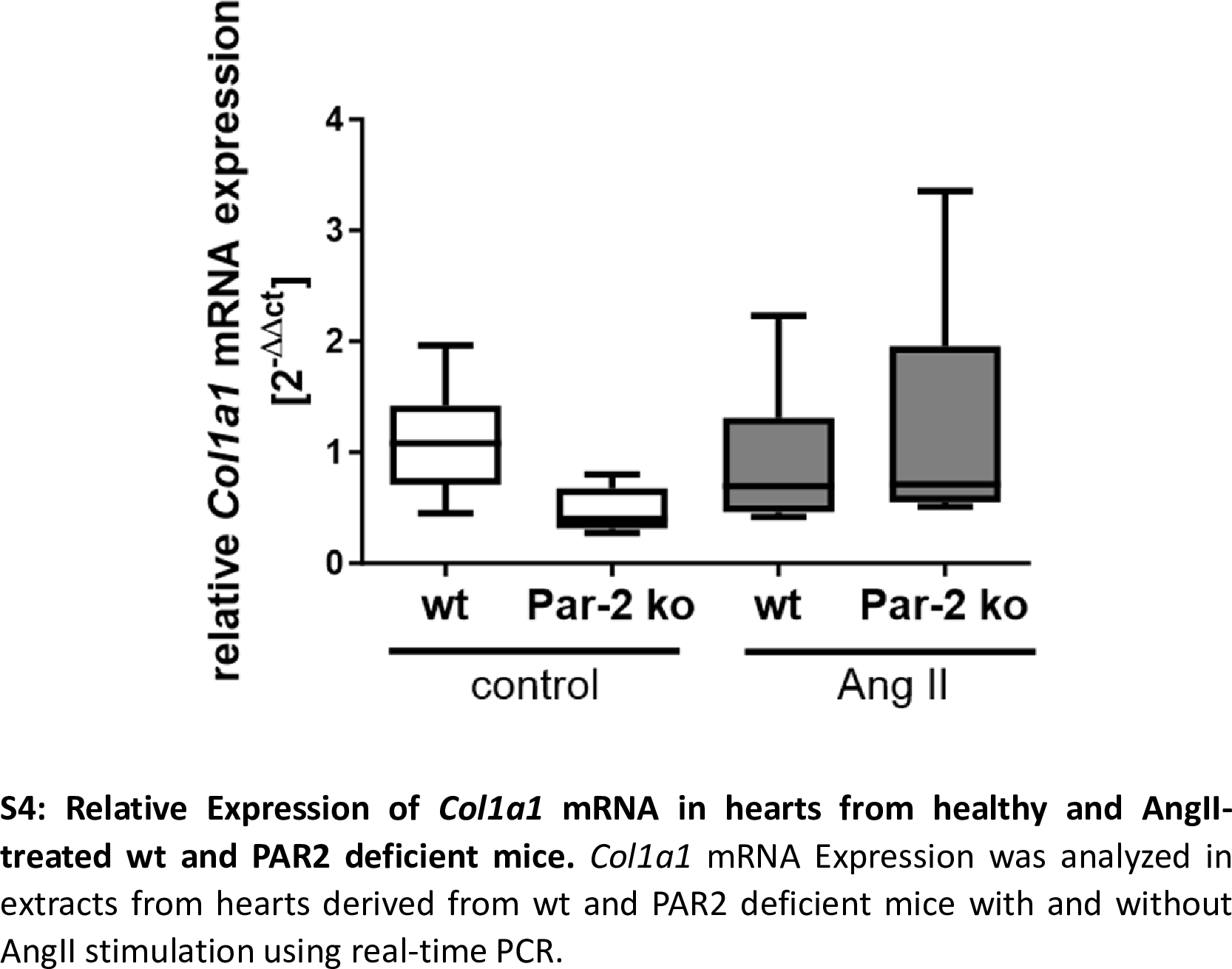

Supplement: S2 Fig — (TIF) [file pone.0310095.s004.tif]

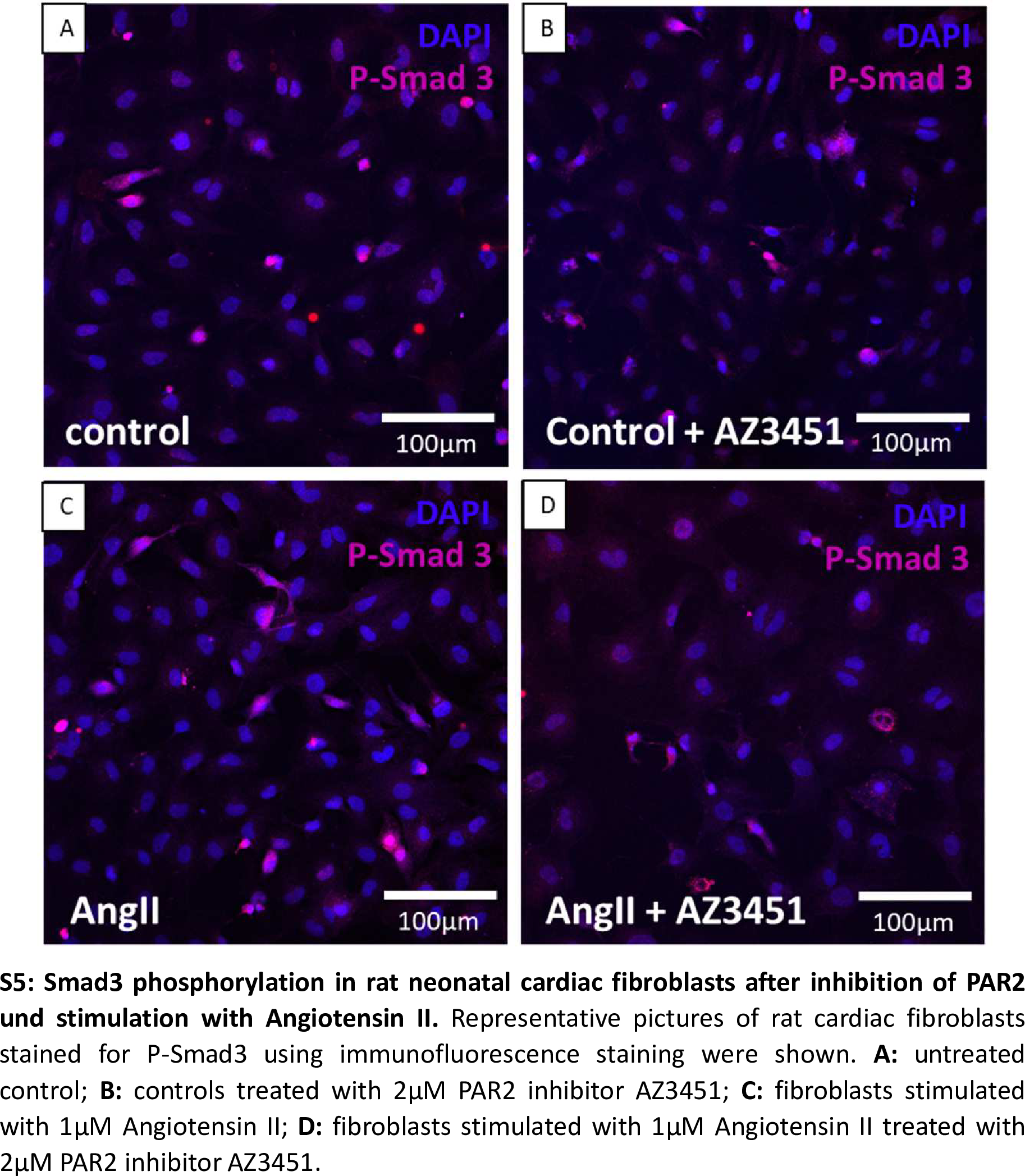

Supplement: S3 Fig — (TIF) [file pone.0310095.s005.tif]
